# Evaluating the influence of land use and land cover change on fine particulate matter

**DOI:** 10.1038/s41598-021-97088-8

**Published:** 2021-09-02

**Authors:** Wei Yang, Xiaoli Jiang

**Affiliations:** 1 School of Geography Science, Taiyuan Normal University, Daxue street, Yuci district, Jinzhong, 030619 Shanxi China; 2 Research Center for Scientific Development in Fenhe River Valley, Taiyuan Normal University, Jinzhong, 030619 Shanxi China

**Keywords:** Environmental sciences, Environmental social sciences

## Abstract

Fine particulate matter (i.e. particles with diameters smaller than 2.5 microns, PM_2.5_) has become a critical environmental issue in China. Land use and land cover (LULC) is recognized as one of the most important influence factors, however very fewer investigations have focused on the impact of LULC on PM_2.5_. The influences of different LULC types and different land use and land cover change (LULCC) types on PM_2.5_ are discussed. A geographically weighted regression model is used for the general analysis, and a spatial analysis method based on the geographic information system is used for a detailed analysis. The results show that LULCC has a stable influence on PM_2.5_ concentration. For different LULC types, construction lands have the highest PM_2.5_ concentration and woodlands have the lowest. The order of PM_2.5_ concentration for the different LULC types is: construction lands > unused lands > water > farmlands >grasslands > woodlands. For different LULCC types, when high-grade land types are converted to low-grade types, the PM_2.5_ concentration decreases; otherwise, the PM_2.5_ concentration increases. The result of this study can provide a decision basis for regional environmental protection and regional ecological security agencies.

## Introduction

With the rapid development of China’s economy and society, its rate of urbanization is accelerating. China’s industrial scale is also expanding rapidly, and the problem of air pollution is becoming increasingly serious, which has a tremendous impact on the environment, economic development, and even people’s health^1^. Fine particulate matter (i.e. particles with diameters smaller than 2.5 microns, PM_2.5_) is considered a crucial protagonist among the various air pollution factors^2^. As a significant health hazard, PM_2.5_ is highly associated with an increased probability of respiratory diseases^3,4^, cardiorespiratory problems^5,6^, mutagenic diseases^7^ and increased mortality. Therefore, it is of vital significance to understand PM_2.5_ pollution clearly, especially its distribution characteristics and influence factors, which are helpful for reducing pollution and protecting human health.

As a severe air pollutant, the concentration of PM_2.5_ is influenced by meteorological factors^8–10^, human activities^11^, and the surrounding environment^12^. PM_2.5_ is emitted mainly from anthropogenic sources, such as from traffic^13^ and industrial production^14^. The spatial and temporal distributions of PM_2.5_ are impacted by meteorological and environment factors^15–17^. Previous research has revealed that PM_2.5_ is severely affected by meteorological factors at the macro-scale^18^ in terms of temperature^19^, precipitation^20^, wind conditions^21,22^, etc., while at the micro-scale, PM_2.5_ is strongly associated with land use and land cover (LULC) type^23^. Optimizing LULC type may reduce PM_2.5_ pollution at the community or city level^24,25^. Land use and land cover change (LULCC) is the embodiment of human activities, which also has an obvious effect on PM_2.5_ distribution^26^. To mitigate pollution, it is significant to explore the effects of LULC and LULCC on PM_2.5_ pollution.

To conduct research on the relationship between LULCC and PM_2.5,_ relevant data are required. Remote sensing based LULCC research has a long history and is relatively mature^27,28^, which has become an effective method to obtain LULCC data. Conventional methods of obtaining PM_2.5_ data employ monitoring stations at fixed sites, whose effective monitoring distances range from 0.5 to 4 km^29^, and which can provide accurate point-source data. The area among the monitoring sites can not been represented by this data. Due to the discontinuous spatial distribution of sites monitoring PM_2.5_ data, several methods have been employed to solve this problem, including spatial interpolation^30^, chemical transport models^31^, land-use regression models^32^ and aerosol optical depth (AOD) based statistical models^33^. However, as the use of a single approach leads to large uncertainties, some researchers have sought to integrate different methods to improve the PM_2.5_ estimation accuracy, such as a combination of chemical transport models and satellite-derived AOD^34,35^.

At present, researches on the relationship between PM_2.5_ and land use mostly focus on city scale^36,37^. Due to atmospheric transport, PM_2.5_ distribution is not only affected by local emissions, but also regional transport^38^. Regional land use changes can directly or indirectly affect PM_2.5_ distribution. There is an insufficient amount on research at regional scale. Moreover, most of the existed researches focus on the influence of landscape patterns on PM_2.5_ pollution but not LULCC types^39,40^. And the PM_2.5_ data used in these studies was station monitoring data which is spatially discontinuous and cannot reveal the spatial relationship between PM_2.5_ and LULCC types. Therefore, in this paper we analyze the relationship between dynamic PM_2.5_ and LULCC type. To avoid the spatial discontinuity of station monitoring PM_2.5_ data, the spatially continuous PM_2.5_ data from the Atmospheric Composition Analysis Group (ACAG) are used. A geographical weighted regression model and a spatial analysis method are employed to identify the response mechanism between dynamic PM_2.5_ and LULCC type. The results of this study can provide a decision basis for regional environmental protection and regional ecological security agencies.

## Methodology

### Study area

**Figure 1:**
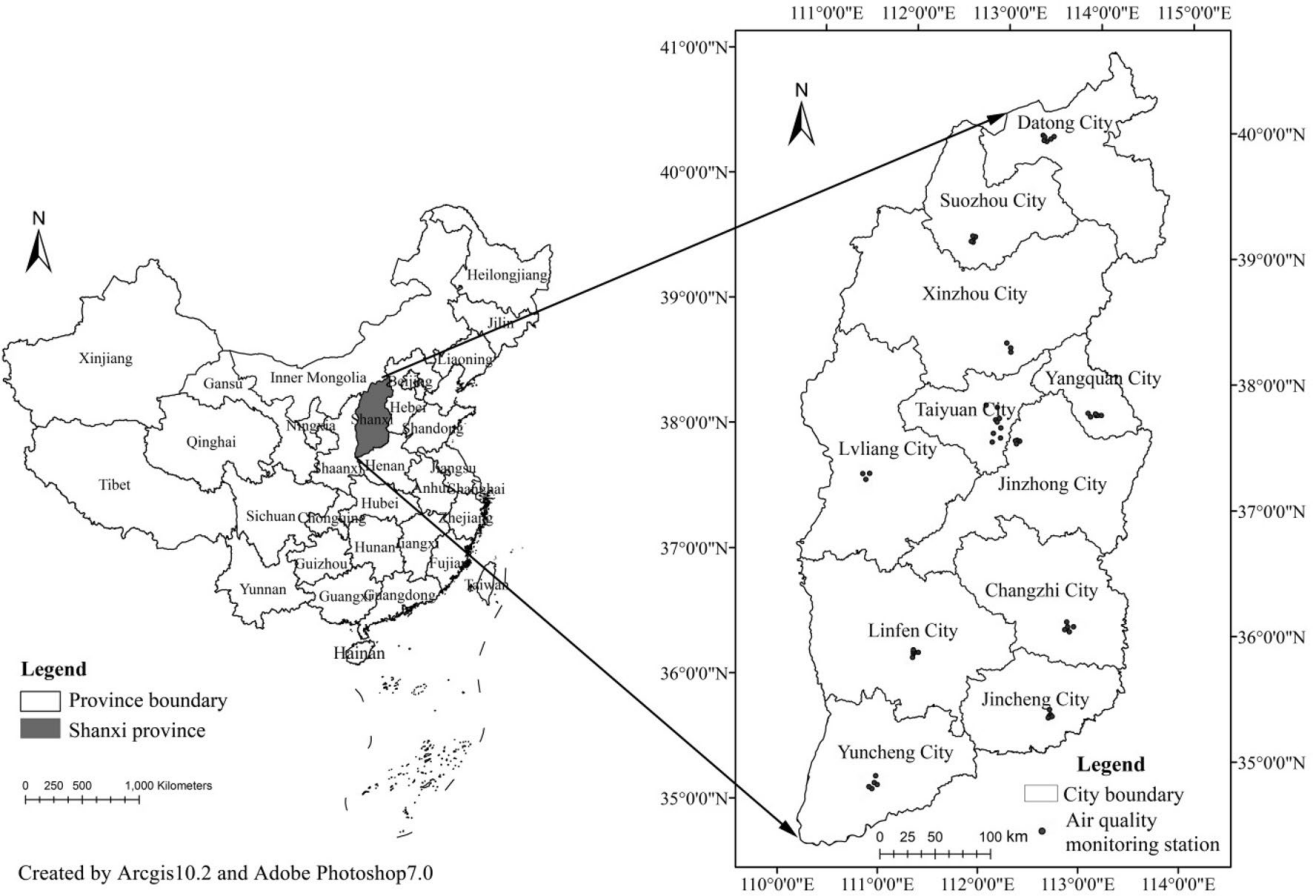
The location of Shanxi Province in China and the location of air quality monitoring stations in Shanxi Province.

Shanxi Province is located in the middle of China (Fig. 1), which is the most important energy bases in the country and whose coal output was ranked first before 2016, and second thereafter. Due to the abundance of coal resources in Shanxi Province, its energy structure is focused on coal, which accounts for 72% of its total energy consumption. Shanxi Province is not only an important coal exporter, but also an important power exporter. The power plants in Shanxi Province are mainly coal-fired, which produce considerable amounts of emissions. Additionally, coking and steel industries are pillar industries in Shanxi Province, which also produce vast amounts of emissions. This economic structure based on energy consumption causes serious air pollution. Several cities in Shanxi Province, such as Taiyuan, Linfen, Jincheng, etc., contain the worst air pollution of all cities in China. Meanwhile, Shanxi Province had experienced obvious LULCCs, such as urban expansion caused by fast urbanization and an increase of green land owing to the growth of the ‘Grain for Green’ project. Therefore, Shanxi Province was selected as the study area to analyze the relationship between LULCC and PM_2.5_.

### Data acquisition and preparation

#### PM_2.5_ data

The PM_2.5_ data provided by ACAG were generated based on a combination of a chemical transport model, satellite observations and ground-based observations^41^. The data have been validated in North America, which have been shown to have higher accuracy than purely geoscience-based estimates^35^. However, the accuracy of the ACAG data of China has not been validated; therefore, in this work we estimated its accuracy (see Sect. 3.1).

**Figure 2:**
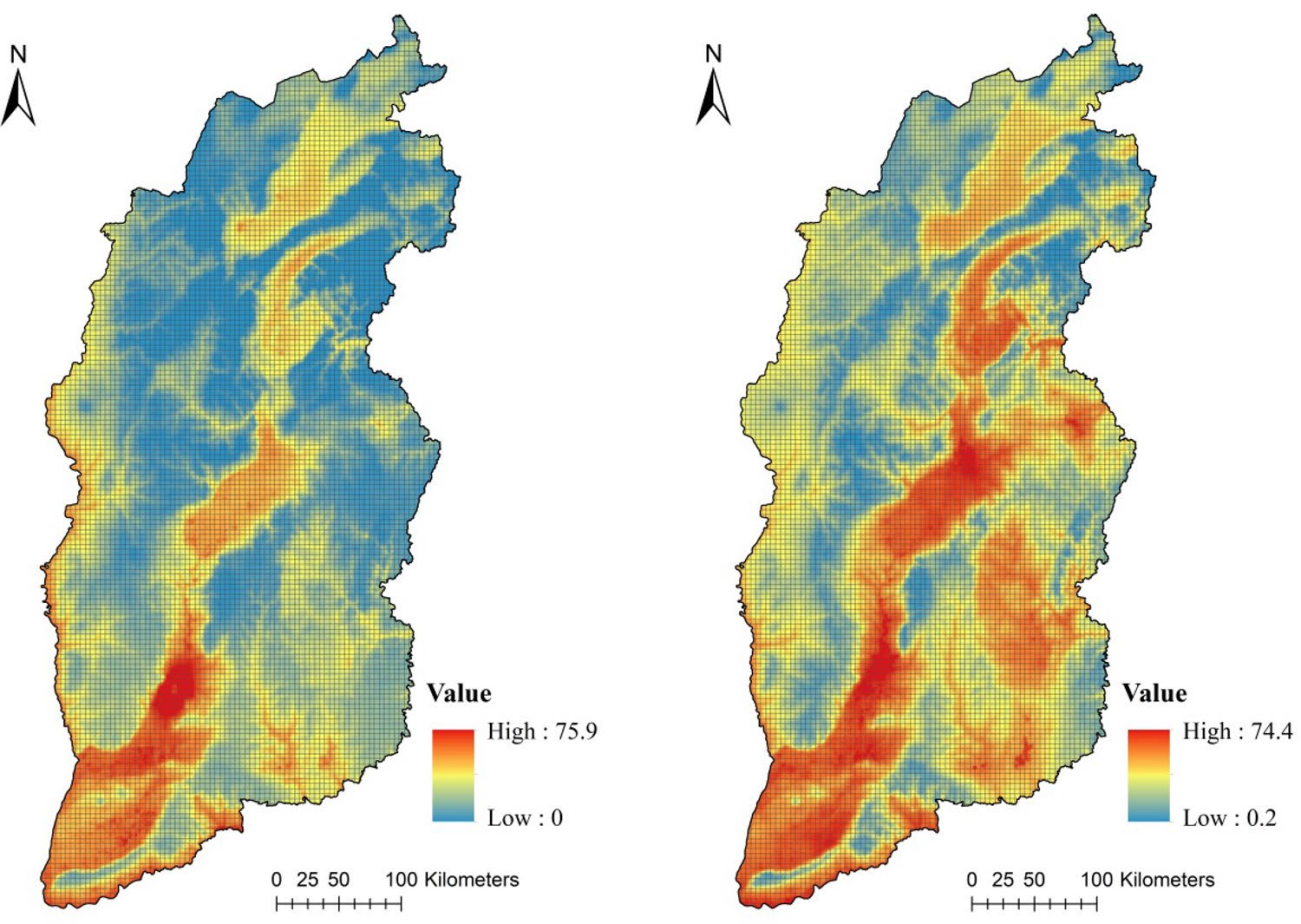
3 × 3 km grid map and ACAG PM2.5 concentration data of Shanxi Province in 2000 and 2018 (units: μg/m^3^).

Ground-based data from 58 state-controlled air quality monitoring stations from 2018 were used in the validation (Fig. 1). The ACAG PM_2.5_ data from 2000 to 2018 were downloaded from (http://fizz.phys.dal.ca/~atmos/martin/?page_id=140%E3%80%822014). The initial data were reprojected and resampled to a 1-km spatial resolution (Fig. 2).

Pearson’s correlation coefficient and the root mean square error (RMSE) were calculated in the validation as:

1 $$\mathrm{Pearson correlation coefficient}=\frac{N\sum {X}_{i}{Y}_{i}-\sum {X}_{i}\sum {Y}_{i}}{\sqrt{N\sum {X}_{i}^{2}-{(\sum {X}_{i})}^{2}}\sqrt{N\sum {Y}_{i}^{2}-{(\sum {Y}_{i})}^{2}}}$$

2 $$\mathrm{RMSE}=\sqrt{\frac{1}{n}\sum_{i=1}^{n}{({X}_{i}-{Y}_{i})}^{2}}$$

where *X*_*i*_ represents a PM_2.5_ value from the ACAG, and *Y*_*i*_ represents a PM_2.5_ value from monitoring stations.

#### Land use and land cover data

**Figure 3:**
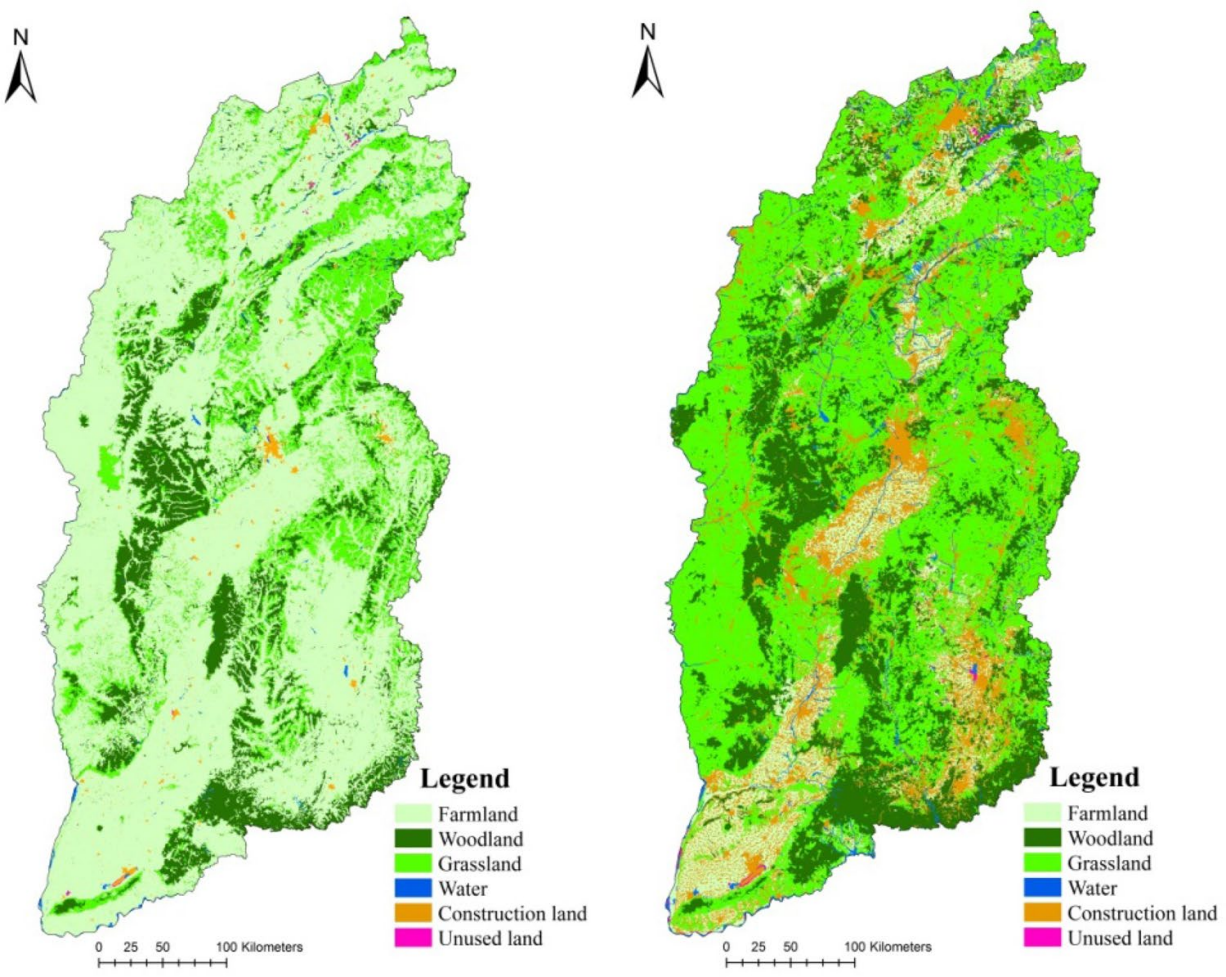
The land use and land cover data in 2000 and 2018.

The China multi-period land use land cover data set (CNLUCC) was used. The CNLUCC data were generated with a visual interpretation method based on Landsat remote-sensing data. The data set was provided by the Data Center for Resources and Environmental Sciences, Chinese Academy of Science (http://www.resdc.cn). Data in 2000 and 2018 were used (Fig. 3). The data consist of six classes: farmlands, forests, grasslands, water, construction lands, and unused lands. The data were shown to have an accuracy of 88.95%, which meet the needs of this study.

### Geographical weighted regression model

Geographical weighted regression (GWR) models are a powerful tool to explore the heterogeneity of spatial relations^42^. As a local spatial regression model, GWR can effectively solve the nonstationarity of variable space, which has been widely used in the spatial analyses of different geographic elements^43^. The essence of GWR is locally weighted least squares, where the ‘weight’ is a distance function of spatial position between the point to be estimated and other observation points. The expression of GWR is as follows:

3 $${y}_{i}={a}_{0}\left({u}_{i},{v}_{i}\right)+\sum_{k}{a}_{k}\left({u}_{i},{v}_{i}\right){x}_{ik}+{\varepsilon }_{i}$$

where *y* is the dependent variable, *x* is the explanatory variable, (*u*_*i*_*, v*_*i*_) is the coordinates of the *i*th point in space, *a*_*k*_(*u*_*i*_*, v*_*i*_) is a realization of the continuous function *a*_*k*_(*u, v*) at point *i*, and ${\varepsilon }_{i}$ is the error term.

To identify the spatial relationship between LULCC and PM_2.5_, a 3 × 3 km grid map (Fig. 2) was generated of the study area. The variations of PM_2.5_ between 2000 and 2018 were calculated in each grid, where the results were considered as the dependent variable in Eq. (3). The changing area of each different land type in each grid was also calculated and considered as the explanatory variable. Four main land types, farmlands, woodlands, grasslands and construction lands, were selected as the explanatory variables because their combined area accounted for nearly 99% of the total area.

### Analysis framework

**Figure 4:**
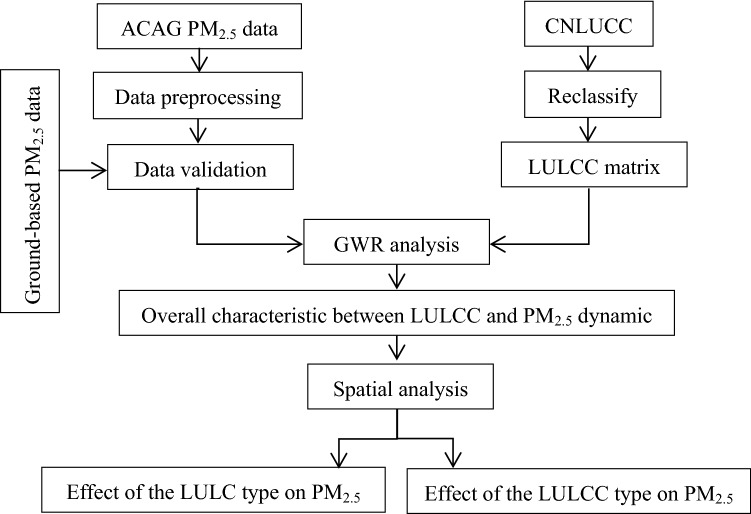
Analysis framework used in this study.

A GWR analysis was used to determine the overall characteristic between the LULCC and PM_2.5_ dynamics. After that, based on the spatial analysis tools in ArcGIS 10.2, a detail analysis was conducted from two aspects: (1) PM_2.5_ distributions for the different LULC types, and (2) PM_2.5_ dynamics for the different LULCC types. The analysis process is shown in Fig. 4.

## Results

### Validation of the PM_2.5_ data

As mentioned above, station monitoring data can represent a scope from 0.5 to 4 km. Thus, a 4-km buffer from each monitoring station was generated. In the buffer, the mean values of the PM_2.5_ data from ACAG were calculated and validated according to the station monitoring data. The results (Table 1) show an RMSE of 7.05 and a Pearson’s correlation coefficient of 0.82, which show that the ACAG PM_2.5_ data have high consistency with the ground-based observational data.

**Table 1:** Validation of the PM_2.5_ data (units: μg/m^3^).

PM_2.5_	Max	Min	Mean	SD	RMSE	Pearson correlation
Station monitoring	84.03	30.42	58.18	12.01	7.05	0.82
ACAG	74.40	32.20	59.78	11.19		

### GWR analysis

**Figure 5:**
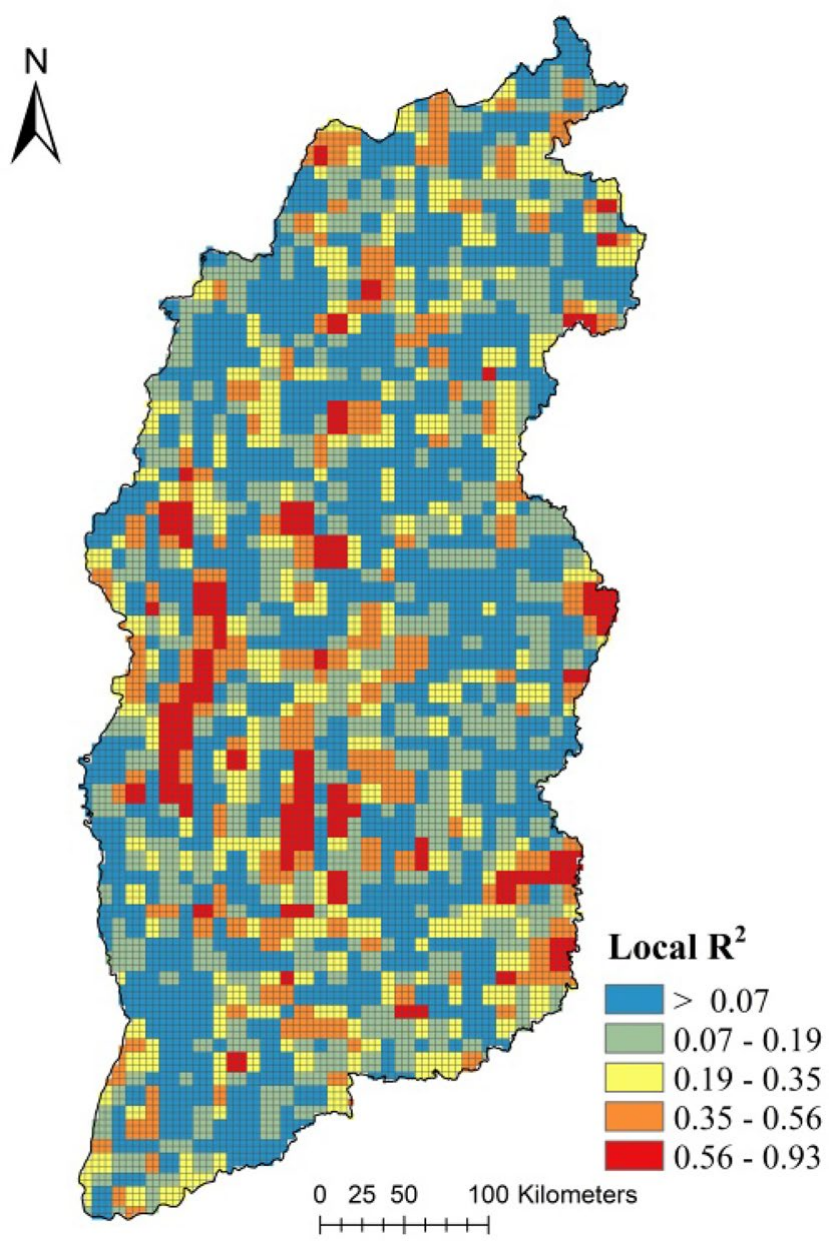
Local R^2^ values from the GWR analysis.

The GWR analysis showed that R^2^ reached 0.94 which implies a good fitting effect. 93.56% of the standardized residuals were between − 2 and 2, which demonstrates that the model fitting was stable^44^. The results show that there was a stable relationship between PM_2.5_ and LULCC. As shown in Fig. 5, the local R^2^ values were between 0.01 and 0.93. In contrast with the LULCC data, the high values of the local R^2^ were distributed in places where the LULCC showed an obvious dynamic while the low local R^2^ values were distributed in LULCC areas that did not change. The result indicated that dynamic PM_2.5_ had a significant response to LULCC.

### Effect of the LULC type on PM_2.5_

To further investigate the relationship between the PM_2.5_ dynamics and the different LULC types, a spatial analyze based on ArcGIS was conducted. The results (Table 2) show that, for all LULC types, the mean PM_2.5_ concentrations significantly increased from 2000 to 2018. Among them, unused lands had the largest increase. Woodlands and grasslands had the largest increasing rates, 86.02% and 81.00%, respectively. Construction lands had the lowest increasing rate of 20.92%. The rates of increase of other the LULC types were relatively close, with a scope of 38.45% and 47.99%. The increasing trends indicate that the PM_2.5_ pollution situation worsened during the study period. The standard deviations (SDs) all increased, meaning that the spatial difference of PM_2.5_ pollution was increased. Indeed, the whole study area is faced with a seriously PM_2.5_ polluted situation.

**Table 2:** PM_2.5_ concentrations of different the LULC types (units: μg/m^3^).

LULC type	2000				2018			
	Min	Max	Mean	SD	Min	Max	Mean	SD
Farmlands	0	75.90	25.34	14.40	1.00	72.90	37.50	14.60
Woodlands	0	62.10	10.87	9.45	0.40	65.70	20.22	11.71
Grasslands	0	60.80	15.21	11.51	0.40	68.90	27.53	12.71
Water	0	72.30	29.78	16.04	1.80	73.10	41.23	15.16
Construction lands	0	75.60	33.92	13.58	1.70	74.40	43.95	14.45
Unused lands	0	68.50	30.21	14.92	2.50	63.80	43.45	14.11

Furthermore, for the different LULC types, in 2000, woodlands had the lowest mean PM_2.5_ concentration, although that of the grasslands was very similar. Construction lands had the highest mean PM_2.5_ concentration. In 2018, the mean PM_2.5_ concentrations of the woodlands and grasslands were still very close, and were still the lowest values. The PM_2.5_ concentration of the construction lands was still the highest. In both 2 years, the order of PM_2.5_ concentration for the different LULC types was the same: construction lands > unused lands > water > farmlands > grasslands > woodlands, meaning that the LULC type had an important influence on the PM_2.5_ concentrations.

### Effect of the LULCC type on PM_2.5_

#### LULCC matrix

As showed in Table 3, in 2000, the main land type in Shanxi Province was farmland, whose area was 6.12 × 10^4^ km^2^, accounting for 39.09% of the total area. Next in total area were grasslands and woodlands, accounting for 29.16% and 28.01% respectively. Construction lands covered 0.42 × 10^4^ km^2^, accounting for 2.67% of the total area. The areas of water and unused lands were very little, accounting for just 0.97% and 0.10% respectively. In 2018, although farmlands still covered the largest area, its area reduced to 5.78 × 10^4^ km^2^, accounting for 36.91% of the total area. The area of woodlands increased, accounting for 28.36% of the total area, and became the second largest land type. The area of grasslands decreased by 0.16 × 10^4^ km^2^ and became the third largest land type. The area of construction lands increased dramatically, and its proportion increased to 5.56%, which was two times greater than in 2000. Water and unused lands still covered very little area, accounting for 0.94% and 0.08% of the total, respectively.

**Table 3:** LULCC in Shanxi Province between 2000 and 2018 (units: km^2^).

2000	2018						Total
	Farmlands	Woodlands	Grasslands	Water	Construction lands	Unused lands	
Farmlands	38,293	5281	12,243	542	4788	41	61,188
Woodlands	4984	31,572	6572	118	584	8	43,838
Grasslands	12,058	7341	24,791	260	1162	25	45,637
Water	574	85	182	507	153	23	1524
Construction lands	1798	98	253	26	2005	4	4184
Unused lands	58	15	19	12	14	31	149
Total	57,765	44,392	44,060	1465	8706	132	156,520

From the perspective of land being converting from one type to another, there was a large conversion of farmlands to other land types, roughly 2.29 × 10^4^ km^2^. Grasslands, woodlands, and construction lands underwent the largest amounts of change, accounting for 53.47%, 23.07%, and 20.91% of the total converted area, respectively. On the other hand, the other land types that were converted to farmlands accounted for 1.95 × 10^4^ km^2^, which significant decreased the total area of farmlands. The main conversion types of woodlands to other land types were grasslands, farmlands and construction lands. The sources of woodlands were mainly grasslands and farmlands. It was seen that the amount of woodlands converted to other types, and those converted to woodlands, were nearly equivalent in total area. Grasslands showed a similar trend as seen for the woodlands, which also showed a relatively stable state. Construction lands were mainly converted to farmlands, which accounted for 82.51% of the total converted area. The total area of construction lands that were converted to other types was 0.22 × 10^4^ km^2^. The main conversion sources of construction lands were farmlands, grasslands, and woodlands, and the total conversion area was 0.67 × 10^4^ km^2^, which was caused by fast urbanization.

#### PM_2.5_ dynamics

As water and unused lands covered only 1% of the total area, we only considered the other four land types (farmlands, woodlands, grasslands, and construction lands) to ascertain the influence of the LULCC types on the PM_2.5_ dynamics. As discussed above, the PM_2.5_ concentrations considerably increased from 2000 to 2018 for all land types, which indicated that there would be a PM_2.5_ concentration increase for non-LULCC areas. The increase was mainly caused by increased pollution levels, not by LULCC. This would bring disturbance to our analysis. To avoid this disturbance, the range of PM_2.5_ concentration variations in non-LULCC areas was calculated first (Table 4) and set as the reference variation range when analyzing the PM_2.5_ concentration variations in the LULCC areas.

**Table 4:** Dynamic PM_2.5_ concentrations in the non-LULCC areas (units: μg/m^3^).

LULCC type	PM2.5 concentration		Reference variation range
	2000	2018	
Farmland to farmland	27.98	40.46	12.48
Woodland to woodland	10.16	18.79	8.63
Grassland to grassland	14.85	27.33	12.48
Construction land to construction land	35.58	50.67	15.09

As showed in Table 4, the PM_2.5_ dynamics in the different LULCC types showed two opposing trends, increasing and decreasing. The largest increase was for woodlands converted to construction lands, while the largest decline was for farmlands converted to woodlands. When farmlands were converted to woodlands and grasslands, the PM_2.5_ concentrations declined, but when they were converted to construction lands, the PM_2.5_ concentration increased. Increasing trends were seen when woodlands were converted to the other three land types. Conversely, declining trends were found when construction lands were converted to the other land types. When grasslands were converted to woodlands, a declining trend was witnessed, but when they were converted to the other two land types, increasing trends were seen.

As discussed above, the PM_2.5_ concentrations for the four land types showed similar trends in both years: construction lands > farmlands > grasslands > woodlands. Therefore, according to the PM_2.5_ concentrations, the four land types were divide into four grades: highest (construction lands), high (farmlands), medium (grasslands) and low (woodlands). As showed in Table 5, when high-grade land types are converted to low-grade types, the PM_2.5_ concentrations decrease, and when low-grade land types are converted to high-grade types, the PM_2.5_ concentrations increase.

**Table 5:** Dynamic PM_2.5_ concentrations in the LULCC areas (units: μg/m^3^).

LULCC type	PM2.5 concentration		Variation range	Reference variation range	Variation trend
	Before change	After change			
Farmland to woodland	15.58	26.11	10.53	12.48	− 1.94↓
Farmland to grassland	18.73	30.74	12.01	12.48	− 0.47↓
Farmland to construction land	31.30	45.52	14.22	12.48	1.74↑
Woodland to farmland	16.00	26.57	10.57	8.63	1.95↑
Woodland to grassland	9.69	21.99	12.30	8.63	3.68↑
Woodland to construction land	16.28	28.81	12.53	8.63	3.90↑
Grassland to farmland	18.76	31.48	12.26	12.48	0.24↑
Grassland to woodland	9.77	21.87	12.09	12.48	− 0.39↓
Grassland to construction land	18.82	33.82	15.01	12.48	2.52↑
Construction land to farmland	33.71	47.32	13.61	15.09	− 1.47↓
Construction land to woodland	20.31	34.48	14.18	15.09	− 0.91↓
Construction land to grassland	20.69	35.25	14.56	15.09	− 0.53↓

## Discussion

As an important energy base, the economic development of Shanxi Province has been mainly based on energy consumption, which continues to generate large quantities of harmful emissions^45^. Therefore, human activities were considered as the most important influence factor of PM_2.5_ pollution^11^. However, LULC types were also representative of different human activities^46^. Different to previous studies, which mainly focused on discussing the relationship between land use type and PM_2.5_ concentrations at urban scales^37,47^, in this study we discussed the impact of land use on PM_2.5_ concentrations from two aspects: different LULC and LULCC types at regional scales.

The different LULC types indicated the different intensities of human activities. Construction lands represented the highest intensity because of the high population density, traffic flow, industrial and commercial activities, etc., therein. All of these generate large quantities of air pollutants and caused the highest PM_2.5_ concentrations^48^. Farmlands were also intensively affected by human activities, which caused relatively high PM_2.5_ concentrations. Firstly, straw burning in farmlands can result in a sharp increase of PM_2.5_ concentration within a short time^49^. Secondly, as a great agricultural country, the use of fertilizer in China is pervasive, and emissions arising from the manufacturing and use of fertilizer have a strong relationship with PM_2.5_^50^. For example, fertilizer liberated from the soil can be converted into a precursor of PM_2.5_^51^. Thirdly, heating activities in rural areas in winter mainly consist of burning coal, which generates large quantities of air pollutants and has an important impact on the PM_2.5_ concentration in farmlands^52,53^. Vegetation covered area, including woodlands and grasslands, had relatively low PM_2.5_ concentrations. These areas were less influenced by human activities, as indicated by the lower pollutant emissions therein. Meanwhile, it has been suggested that vegetation coverage has a negative regulating effect on PM_2.5_ concentration^54,55^. Thus, woodlands have the lowest PM_2.5_ concentrations because of their highest vegetation coverage.

The different LULCC types represented transitions among the different intensities of human activities, which caused dynamic changes of the PM_2.5_ concentrations. When other land types were converted to construction lands, the intensity of human activities increased, which caused an increase of PM_2.5_ concentration. A similar conclusion was found in another study, which showed that when natural land cover is replaced by manmade areas PM_2.5_ concentrations increase^56^. Furthermore, other LULCC types were also discussed in our study. Farmlands may also contain intense human activities that can increase the PM_2.5_ concentration, such as agricultural activities^57,58^. This was demonstrated by the increasing trend of PM_2.5_ concentration when woodlands and grasslands were converted to farmlands. As vegetation coverage had a negative effect on PM_2.5_ concentration^55^, the PM_2.5_ concentration also changed when the vegetation type changed; i.e. an increase trend was seen when woodlands were converted to grasslands.

Due to the limited LULC data, this study illustrated the influence of LULC and LULCC on PM_2.5_ at the regional scale where human activities were considered as the most important influence factor. However, PM_2.5_ pollution is both affected by human and natural factors^59^. In desert areas, natural factors including dust and wind could be the most important factors^60^, while in coastal areas, climatic elements had the most important influence on PM_2.5_ pollution^37^. These situations were not discussed in the present study. Future studies at larger scales are required to demonstrate the influence of LULC on PM_2.5_ more comprehensively. The relationship between PM_2.5_ pollution and its influence factors is complex and non-linear^61^. Traditional linear analysis methods have certain limitations and new non-linear analysis methods should be employed. Moreover, higher spatial and temporal resolution PM_2.5_ data and LULC data are also required to better understand the response mechanism of PM_2.5_ pollution to LULCC.

## Conclusions

In this study, high-accuracy PM_2.5_ data from ACAG and LULC data were employed to explore the relationship between PM_2.5_ and LULCC. A GWR method was used for the general analysis, and a spatial analysis method based on the geographic information system was used for the detailed analysis. The main conclusions can be drawn as follows:

1. The GWR analysis showed that R^2^ reached 0.92, which represented a stable relationship between PM_2.5_ and LULCC. High local R^2^ values located in highly dynamic LULCC areas indicated that the dynamic PM_2.5_ had a significant response to LULCC.
2. In both considered years, 2000 and 2018, the order of PM_2.5_ concentration in the different LULC types was the same: construction lands > unused lands > water > farmlands >grasslands > woodlands, meaning that the LULC type had an important influence on the PM_2.5_ concentration.
3. LULCC can also impact the dynamics of PM_2.5_ concentration. When low-grade land types are converted to high-grade types, the PM_2.5_ concentration increases; otherwise, it decreases. From another angle, when natural lands are converted to human-related lands, the PM_2.5_ concentration increase; otherwise, the PM_2.5_ concentrations decrease.
